# Next-Generation Eco-Friendly Hybrid Air Purifier: Ag/TiO_2_/PLA Biofilm for Enhanced Bioaerosols Removal

**DOI:** 10.3390/ijms26104584

**Published:** 2025-05-10

**Authors:** Rotruedee Chotigawin, Bhuvaneswari Kandasamy, Paradee Asa, Tistaya Semangoen, Pravech Ajawatanawong, Sarun Phibanchon, Taddao Pahasup-anan, Surachai Wongcharee, Kowit Suwannahong

**Affiliations:** 1 Faculty of Public Health, Burapha University, Chonburi 20131, Thailand; rotruedee@go.buu.ac.th (R.C.); bhuviphy11@gmail.com (B.K.); paradeeasa@go.buu.ac.th (P.A.); taddao.pa@go.buu.ac.th (T.P.-a.); 2 Department of Civil Engineering, Faculty of Engineering, Rajamangala University of Technology Thanyaburi, Pathum Thani 12110, Thailand; 3 Department of Medical Technology, Faculty of Allied Health Sciences, Burapha University, Chonburi 20131, Thailand; tistaya@go.buu.ac.th; 4 Division of Medical Bioinformatics, Research Department, Faculty of Medicine Siriraj Hospital, Mahidol University, Bangkok 10700, Thailand; pravech.aja@mahidol.ac.th; 5 Department of Innovation and Educational Technology, Burapha University, Chonburi 20131, Thailand; sarunp@go.buu.ac.th; 6 Field of Environmental Engineering, Faculty of Engineering, Mahasarakham University, Mahasarakham 44150, Thailand; surachai.w@msu.ac.th

**Keywords:** air pollution, antibacterial activity, bioaerosols, Ag nanoparticle, TiO_2_ nanoparticles, hybrid air purifier

## Abstract

Indoor air pollution poses a significant public health risk, particularly in urban areas, where PM2.5 and airborne contaminants contribute to respiratory diseases. In Thailand, including Chonburi Province, PM2.5 levels frequently exceed safety thresholds, underscoring the urgent need for effective mitigation strategies. To address this challenge, we developed a hybrid air purification system incorporating a bioplastic-based photocatalytic film of polylactic acid (PLA) embedded with titanium dioxide (TiO_2_) nanoparticles. For optimization, PLA films were functionalized with varying TiO_2_ concentrations and characterized using SEM, FTIR, TGDTA, and UV–Vis. spectroscopy. A 5 wt% TiO_2_ loading was identified as optimal and further enhanced with silver (Ag) nanoparticles to boost photocatalytic efficiency. The Ag/TiO_2_/PLA biofilm was fabricated via a compound pellet formulation process followed by blown film extrusion. Various compositions, with and without Ag, were systematically evaluated for photocatalytic performance. The novel customized hybrid air purifier developed in this study is designed to enhance indoor air purification efficiency by integrating Ag/TiO_2_/PLA biofilms into a controlled oxidation system. The air purification efficacy of the developed biofilm was evaluated through a controlled study on *Staphylococcus aureus* (*S. aureus*) removal under different treatment conditions: control, adsorption, photolysis, and photocatalytic oxidation. The impact of light intensity on photocatalytic efficiency was also examined. The photocatalytic oxidation of *S. aureus* was subjected to the first-order kinetic evaluation through mathematical modeling. Results demonstrated that the Ag/TiO_2_/PLA biofilm significantly enhances indoor air purification, providing a sustainable, scalable, and energy-efficient solution for microbial decontamination and pollutant removal. This innovative approach outperforms conventional adsorption, adsorption and photocatalytic oxidation systems, offering a promising pathway for improved indoor air quality.

## 1. Introduction

Environmental factors such as temperature, humidity, and airflow significantly impact health. High temperatures cause discomfort and sweating, while cold temperatures lead to vasoconstriction and shivering. Humidity extremes hinder sweat evaporation or cause skin and respiratory irritation. Poor air circulation allows airborne pollutants and microorganisms to accumulate, increasing health risks. Fine particulate matter (PM2.5) acts as a carrier for bacteria and endotoxins, further exacerbating respiratory conditions [1]. Other key pollutants, such as carbon monoxide (CO), sulfur dioxide (SO_2_), nitrogen dioxide (NO_2_), and volatile organic compounds (VOCs), also contribute to asthma, bronchitis, and lung cancer [2,3,4]. Radon gas in adsorption and ozone (O_3_) emissions from office equipment further degrades indoor air quality. Thailand, including Chonburi Province, is experiencing dangerously high PM2.5 levels, primarily from vehicle emissions, agricultural burning, and industrial activities [5,6]. These ultrafine particles infiltrate indoor environments, promoting bioaerosol formation and bacterial proliferation [7]. The combination of bacterial contamination and particulate pollution significantly increases the risk of respiratory diseases [8]. Airborne bacteria, including *S. aureus*, Legionella pneumophila, and Pseudomonas aeruginosa, can spread through air conditioning systems and inadequate ventilation, causing respiratory infections such as pneumonia and Legionnaire’s disease [9]. Eliminating *S. aureus* is crucial due to its role in antibiotic resistance, infections, and contamination risks [10]. It causes conditions ranging from skin infections to life-threatening sepsis, with methicillin-resistant strains (MRSAs) posing significant treatment challenges. Beyond healthcare, *S. aureus* threatens food safety by producing toxins that cause food poisoning [11]. In hospitals and industries, it contaminates surfaces and medical devices, increasing infection risks [12]. Effective removal methods, including disinfectants, biological treatments, and nanotechnology, are essential for preventing its spread and ensuring public health safety [13]. Effective air quality management requires proper ventilation, pollutant control, and antimicrobial strategies to mitigate bacterial exposure and enhance overall well-being [14,15].

Studies have shown that microorganisms can remain suspended in the air for days or even months, significantly impacting indoor air quality [16]. Research in Thailand and internationally has focused on monitoring airborne microorganisms, particularly in hospitals [15,17]. A study in South Korea (Park, D. et al., 2013) found airborne bacteria levels ranging from 200 to 870 CFU/m^3^ across six hospitals [18]. In Thailand, a 2008 study measured an average of 493 CFU/m^3^ in a Khon Kaen public hospital [18].

To combat bioaerosols, various engineering solutions have been implemented, including ventilation systems designed to meet ASHRAE standards and high-efficiency particulate air (HEPA) filters capable of eliminating 99.97% of airborne microorganisms [19]. Additional methods, such as UV control, ozone systems, and disinfectant sprays, have also proven effective [19,20]. Air purifiers have gained attention as a practical solution for reducing airborne microorganisms. Photocatalytic oxidation technology has demonstrated efficiency rates of 76–98% [21,22]. This method is gaining popularity due to its non-toxic nature, affordability, and ability to operate at room temperature [23]. The photocatalytic oxidation process activates semiconductors under UV radiation, generating powerful oxidizing agents that break down organic matter into water and carbon dioxide. Among the various photocatalysts, TiO_2_ is widely used due to its safety, affordability, stability, and reusability while maintaining high efficiency [24]. To enhance treatment efficiency, heavy metals such as silver and copper are often incorporated into TiO_2_, improving its catalytic properties [22,25]. However, TiO_2_ poses environmental challenges after its effective lifespan expires, necessitating research into sustainable disposal or regeneration methods [26,27].

Building upon existing research, this study aims to develop a hybrid air purifier utilizing photocatalytic oxidation in combination with a bioplastic film to eliminate bioaerosols. This research is innovative, as no prior studies have explored this specific approach. The core of this development lies in the creation of a biodegradable bioplastic film derived from plant-based materials, such as corn husks. This environmentally friendly material [4] is coated with TiO_2_ and integrated into an air purification system to enhance bioaerosol elimination.

This research mainly focuses on developing an eco-friendly, efficient, and cost-effective air purification system by evaluating the efficiency of air purifiers in removing bioaerosols through the integration of bioplastic films and photocatalytic oxidation. This investigation employed an experimental approach, consisting of three primary stages: 1. Preparation of different dosages of TiO_2_-loaded PLA-based biofilm and Ag/TiO_2_/PLA-enhanced bioplastic films with optimized photocatalytic capabilities. 2. The physical properties of these bioplastic films were thoroughly analyzed to determine their viability for air purification. 3. Furthermore, the effectiveness of air purifiers utilizing these films in eliminating bioaerosols was rigorously assessed to explore their potential for enhancing indoor air quality.

These PLA-based bioplastic films, enhanced with Ag/TiO_2_, are produced through a blow film process, where they are heated to between 100 and 190 °C and shaped into thin sheets using air-blowing techniques. Their properties are analyzed using Scanning Electron Microscopy (SEM) to assess particle distribution. This study also explores how different types of bioplastic films and varying light intensities over time influence air purifier efficiency in bioaerosol elimination. In particular, this study investigated the efficacy of air purifiers in eliminating *S. aureus*, considering various factors. The experiment was conducted under controlled conditions, with a temperature range of 25–27 °C and continuous air circulation. The air circulation rate was set to 5 L/min to simulate typical indoor ventilation conditions. To ensure accuracy, the experimental setup was thoroughly cleaned and disinfected before and after each trial. The results are presented in four key areas: utilizing biodegradable materials, this research aims to reduce environmental pollution, decrease reliance on synthetic plastics, and promote the development of cost-effective, sustainable air purification solutions. Ultimately, these advancements could lead to cleaner indoor environments, improved health outcomes, and broader environmental sustainability.

## 2. Results

### 2.1. SEM Analysis of S. aureus

**Figure 1:**
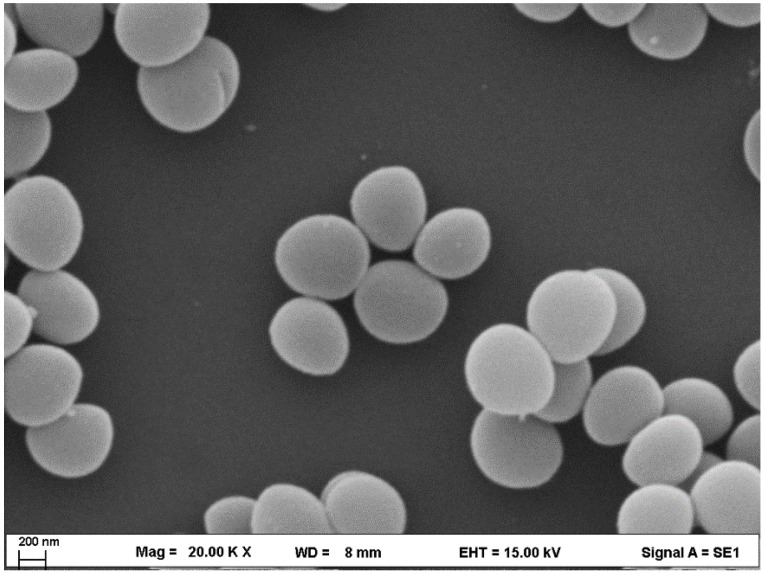
SEM image of *S. aureus*.

The SEM image provides a high-resolution visualization (20,000× magnification) of *S. aureus*, a Gram-positive bacterium known for its pathogenic potential and role in human infections (Figure 1). The image demonstrated a bacterial morphology in cocci arranged in clusters, which is one of the features of the genus *Staphylococcus*. The cells are smooth and well-defined, without visible external flagella that describes non-motile of *S. aureus*. The clustering of the cells suggests active cell division, as *S. aureus* divides in multiple planes. The arrangement may also indicate early-stage biofilm formation, a critical factor in its survival on biotic and abiotic surfaces.

### 2.2. SEM Analysis of Developed Biofilms

**Figure 2:**
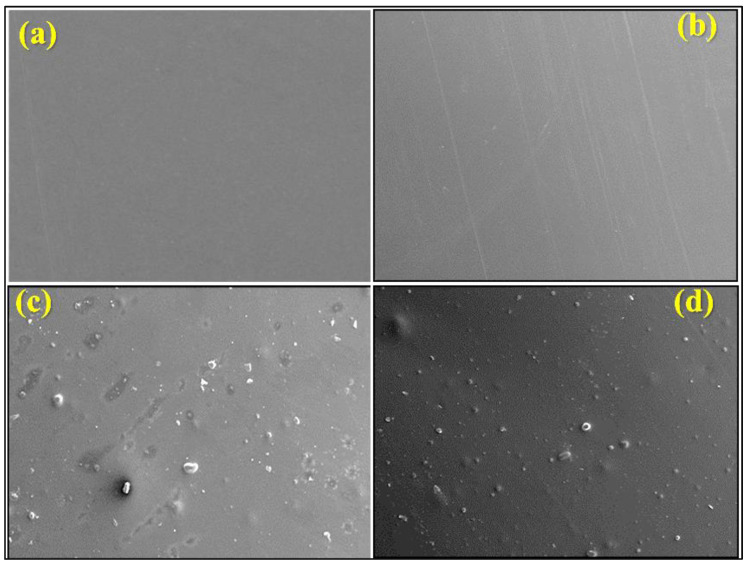
SEM images of developed bioplastic films: (**a**) B1, (**b**) B2, (**c**) B3, and (**d**) B4.

SEM analysis was employed to investigate the morphology of various bioplastic films. The surface morphology and nanoparticle distribution of different weight % of TiO_2_ nanoparticle-loaded PLA films are shown in Figure 2. At an optimal concentration of 5 wt%, SEM images revealed a uniform dispersion of TiO_2_ nanoparticles with minimal agglomeration, ensuring effective surface coverage and maximum light absorption (Figure 2b). This uniformity enhances photocatalytic efficiency by increasing active sites for photocatalytic reactions and reactive oxygen species (ROS) generation.

Our previous findings showed that excessive TiO_2_ accumulation caused shadowing effects, reducing light absorption and promoting electron–hole recombination, which lowered photocatalytic activity. Dense TiO_2_ clusters also compromised film flexibility and mechanical stability, limiting practical use. The 5 wt% TiO_2_-PLA biofilms achieved the best balance between nanoparticle dispersion, light absorption, and mechanical integrity, making them optimal for air purification. Therefore, 5% TiO_2_ loading was selected for air purifier preparation [28].

Notably, the bioplastic film without semiconductor additives exhibits a uniform surface (Figure 2a), while the film incorporating Ag nanoparticles displays a dispersed Ag distribution (Figure 2b). Conversely, the film containing 5% TiO_2_ by weight reveals a homogeneous TiO_2_ distribution, although increased concentrations lead to agglomeration (Figure 2c). Interestingly, the bioplastic film combining TiO_2_ and Ag NPs exhibits a well-distributed morphology without agglomeration, akin to the TiO_2_-containing film (Figure 2d).

### 2.3. Removal of S. aureus by the Hybrid Air Purifier

This study examined the removal of *S. aureus* from the air under different environmental conditions to evaluate the effectiveness of photocatalytic oxidation in air purification. Four treatment methods were analyzed: control conditions, adsorption, photolysis, and photocatalytic oxidation. Under control conditions, bacterial survival in the air was assessed without external treatment. Adsorption physically removed airborne bacteria based on particle size but did not neutralize them, risking potential re-release. Photolysis exposed bacteria to UV radiation (without a photocatalyst), with efficiency depending on light intensity, wavelength, and exposure time, where UV-C was particularly effective in damaging cell structures. Photocatalytic oxidation, using an advanced photocatalyst under light exposure, generated reactive oxygen species (ROS) like superoxide anions (O_2_^−^), hydroxyl radicals (^•^OH), and hydrogen peroxide (H_2_O_2_), leading to bacterial degradation [21,29]. This method outperformed photolysis by utilizing oxidation-driven disinfection. This study compared these methods to identify the most effective air purification strategy, considering environmental factors such as light intensity, air composition, humidity, and catalyst properties.

### 2.4. Removal of S. aureus by Control Reaction

**Figure 3:**
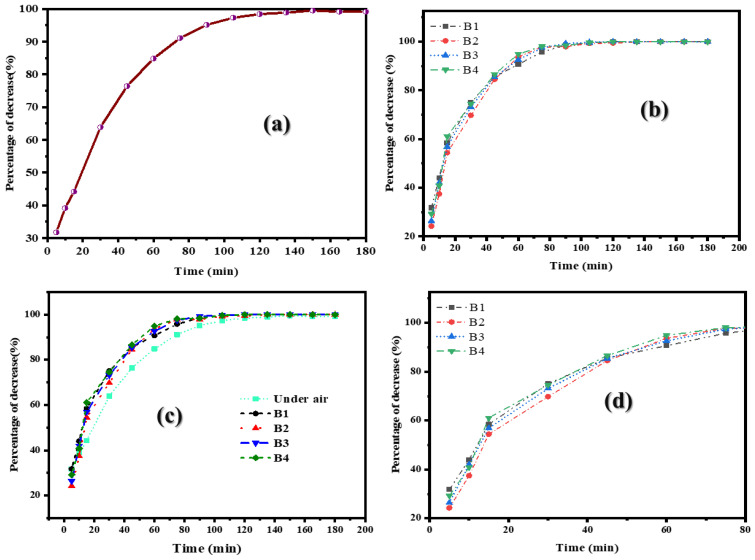
Trend of *S. aureus* reduction via adsorption: (**a**) Control condition, (**b**) Adsorption (**c**) Comparison of S. aureus reduction among bioplastic films and natural reduction, and (**d**) Zoomed-in image of “(**c**)”.

The experiment was conducted in a controlled environment with a consistent temperature range and continuous air circulation. To ensure the precision of the results, the experimental setup was thoroughly sanitized before and after each trial. Initially, this experiment investigated the natural sedimentation of *S. aureus* over a 3 h period. The results showed that, within the initial 10 min, the reduction in *S. aureus* was relatively low. However, as the experiment progressed, the rate of reduction increased significantly. After 75 min, the reduction in *S. aureus* exceeded 90%, indicating that natural sedimentation can be an effective means of reducing airborne microorganisms (Figure 3a).

### 2.5. Adsorption-Based Reduction in S. aureus Through as-Prepared Biofilms

This experiment investigated the efficacy of as-developed bioplastic films in reducing airborne *S. aureus* through adsorption (Figure 3b). The experiment was conducted without UV-C lamp activation. This study found that, within the initial 10 min, the *S. aureus* reduction rate ranged from 31.82% to 44.00%. As the experiment progressed, the reduction rate increased, particularly after the 60-minute mark. Notably, the bioplastic film achieved over 90% bacterial reduction, with 100% reduction observed between 120 and 180 min. Figure 3b shows the observed reduction in *S. aureus* by the physical adsorption mechanism of the as-developed bioplastic film, which captures airborne bacteria through size exclusion and electrostatic attraction. For B1, the initial reduction rate of 31.82–44.00% within the first 10 min suggests a rapid capture of larger bacterial aggregates, while the subsequent increase in reduction rate indicates the capture of smaller bacterial particles. The 100% reduction observed between 120 and 180 min suggests that the PLA bioplastic film is highly effective in removing airborne *S. aureus*. This study revealed that the B2 bioplastic film exhibited a notable reduction in *S. aureus*. Within the initial 15 min, the reduction rate ranged from 24.23% to 54.40%. As the experiment progressed, the reduction rate increased significantly, particularly after the 60-minute mark. Notably, the B2 bioplastic film achieved over 90% bacterial reduction, with 100% reduction observed between 135 and 180 min. The addition of TiO_2_ to the bioplastic film further enhanced its antimicrobial properties, due to its high UV light absorption capacity, as these materials have been shown to exhibit antimicrobial activity against a range of microorganisms. This study suggests that the bioplastic film mixed with TiO_2_ is highly effective in eliminating airborne *S. aureus*, offering implications for developing antimicrobial air adsorption systems. The antimicrobial efficacy of bioplastic film incorporating B4 biofilm showed a notable reduction in bacterial counts, with a 29.19–40.84% decrease within the initial 10 min, followed by a significant increase in reduction rate, achieving over 90% reduction after 60 min and 100% reduction between 120 and 180 min. The rapid initial decline in bacterial count suggests the efficient entrapment of larger bacterial clusters, while the sustained reduction over time indicates the gradual adsorption of smaller particles. The observed antimicrobial activity can be credited to the combined effects of TiO_2_-generated reactive oxygen species under UV light and Ag NPs’ bactericidal properties, which likely disrupted the bacterial cell membrane, ultimately leading to their inactivation. Also, the combination of these two antimicrobial agents, such as Ag and TiO_2_, in a bioplastic matrix (B4) creates a synergistic effect, enhancing bacterial reduction more effectively than either agent alone. This study highlights the potential of as-developed B4 bioplastic as an effective antimicrobial air adsorption system.

#### Comparison of *S. aureus* Reduction Among Bioplastic Films and Natural Reduction

A comparative analysis of the percentage of *S. aureus* reduction among the four types of bioplastic films and natural reduction revealed a similar trend and reduction pattern across all bioplastic films. However, the adsorption efficiency of all bioplastic films exhibited a slightly enhanced bacterial reduction compared to natural reduction, particularly during the 5–120 min period, as illustrated in Figure 3c,d.

This finding suggests that the bioplastic films possess a moderate to high level of adsorption efficiency, which can be attributed to their physical and/or chemical properties that facilitate the capture and/or inactivation of airborne *S. aureus*.

### 2.6. Removal of S. aureus in Air via UV-C Photolysis

**Figure 4:**
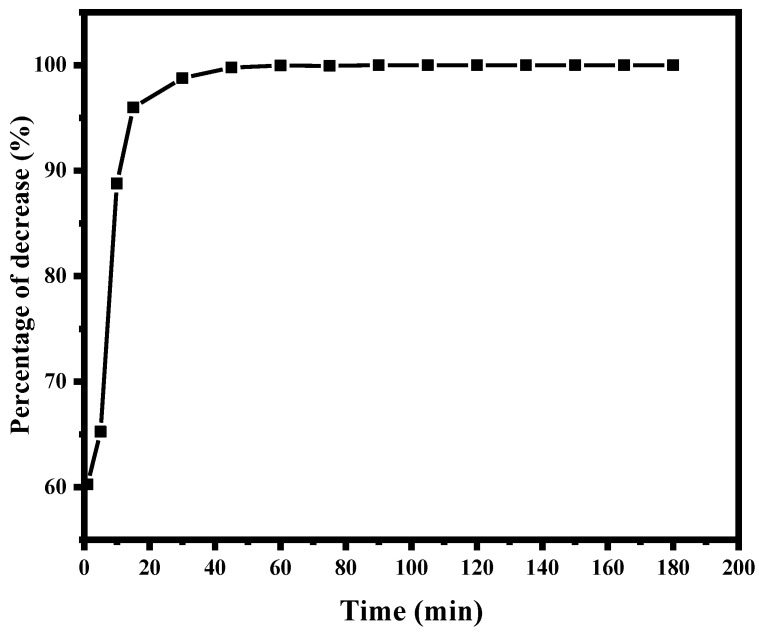
Trend of *S. aureus* concentration in air during the photolysis process (without catalyst).

This study investigated the effectiveness of UV-C photolysis (without photocatalyst) in reducing airborne *S. aureus* using a single UV-C light tube with a wavelength of 254 nanometers. The results demonstrated a significant reduction in bacterial counts, with a 60–65% decrease within the initial 5 min. As the exposure time increased, the reduction rate accelerated, achieving up to 95.99% reduction after 15 min. Notably, complete inactivation of *S. aureus* was observed after 90 min of UV-C exposure (Figure 4).

The observed inactivation of *S. aureus* can be attributed to the damaging effects of UV-C radiation on bacterial DNA, leading to the disruption of important cellular functions and, ultimately, cell death (1). The rapid reduction in bacterial counts within the initial 5 min suggests a high susceptibility of *S. aureus* to UV-C radiation. The complete inactivation of *S. aureus* after 90 min of exposure highlights the potential of UV-C photolysis as an effective method for controlling airborne bacterial populations.

### 2.7. Photocatalytic Oxidation (PCO) Reaction on Bioplastic Film for S. aureus Reduction

This study evaluates the impact of varying TiO_2_ and Ag nanoparticles concentrations in bioplastic films on the reduction in airborne *S. aureus* under UV-C light exposure. The photocatalytic oxidation (PCO) reaction, induced by UV-C activation, involves ROS that interact with bacterial cells, leading to their inactivation.

Bioplastic films with and without the addition of Ag and TiO_2_ were tested at distances of 1 cm and 3 cm from the UV-C light source. For the pure PLA(B1) film, bacterial reduction reached 70% within 30 min and exceeded 90% after 45 min. Similarly, silver-infused bioplastic films (B2) were evaluated; *S. aureus* reduction was around 80% within 30 min and complete removal at 75 min. With the addition of TiO_2_, the reduction rate was significantly enhanced, achieving 92% in 10 min and complete inactivation at 75 min (B3). The B4 biofilm demonstrated the most effective antibacterial activity, with over 92% reduction within 10 min, 99% at 15 min, and total inactivation within 60 min. Bioplastic films containing both TiO_2_ and Ag demonstrated synergistic effects, enhancing bacterial reduction. 

Comparative analysis with natural reduction and adsorption alone showed that these treatments were significantly slower. Under natural conditions, *S. aureus* levels decreased by 40% within 10 min and over 90% at 75 min. Adsorption alone provided minimal bacterial reduction, while photolysis with UV-C achieved 60–65% inactivation within 5 min and complete removal after 90 min. This study highlights the effectiveness of TiO_2_ and Ag bioplastic films in air purification, particularly in high-risk environments. Future research should focus on optimizing the TiO_2_-Ag ratio, assessing film durability, and exploring real-world applications in hospitals, public areas, and air purification devices. Investigating alternative photocatalysts and light sources could further enhance air purification efficiency and sustainability.

**Figure 5:**
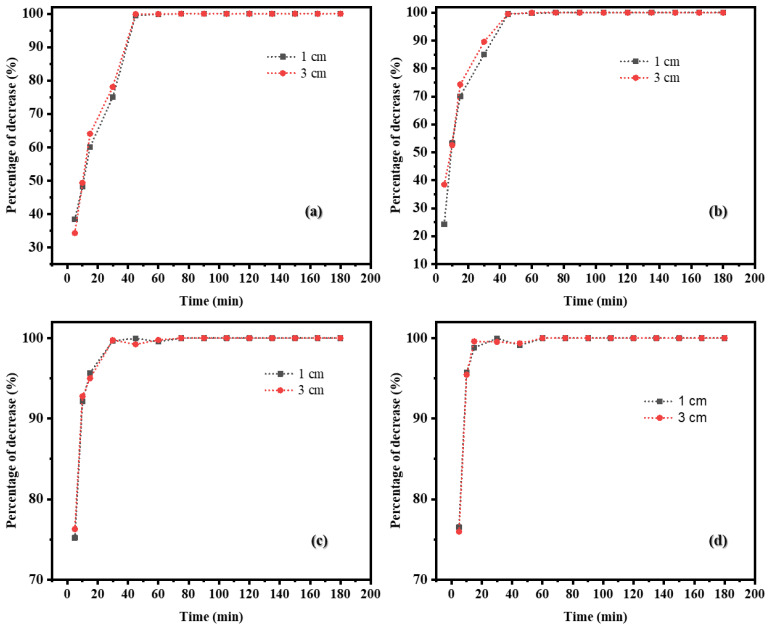
Reduction in *S. aureus* in air using (**a**) B1 and (**b**) B2, (**c**) B3, and (**d**) B4 bioplastic films at 1 cm and 3 cm from a UV-C light source.

**Figure 6:**
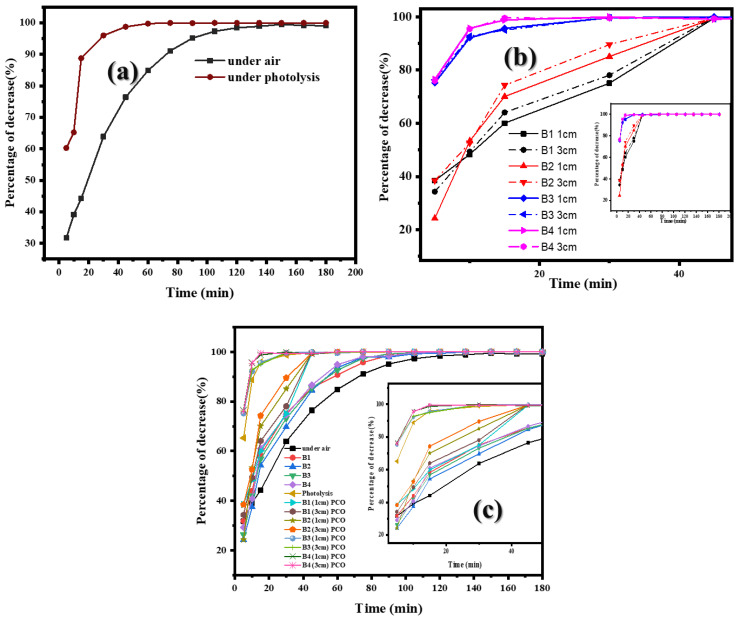
Comparative analysis of *S. aureus* reduction trends: (**a**) air vs. photolysis process, (**b**) UV-C light-assisted PCO process at 1 cm vs. 3 cm distances, and (**c**) control, adsorption, photolysis, and PCO.

The study found that the reduction in bacteria was similar across all types of bioplastic films, regardless of the lamp distance (1 cm and 3 cm) or the photolysis process (Figure 5). When comparing the reduction in bacteria with different films combined with UV-C, it was found that the reductions in bacteria using the bioplastic film mixed with TiO_2_ and the bioplastic film mixed with TiO_2_ and Ag NPs had the highest efficiency and were almost the same, with all bacteria being reduced within 30 min. In addition, when comparing the bioplastic film under PCO condition with the photolysis process, it was found that the efficiency of the reduction in bacteria using the bioplastic film mixed with TiO_2_ and the bioplastic film mixed with TiO_2_/Ag NPs in the air purifier was better than the photolysis process alone, as shown in Figure 6.

The demand for air purifiers to control airborne bioaerosols has increased, particularly during the COVID-19 pandemic. This study focused on developing an air purifier that utilizes photocatalytic oxidation to remove *S. aureus*. All the as-developed bio-films were tested, and these films, combined with UV-C light of varying intensities, were evaluated for three hours.

Under natural conditions, *S. aureus* levels declined by approximately 40% within 10 min and by over 90% after 75 min, aligning with the results of prior studies. The bioplastic films reduced *S. aureus* by 20–50% within the first 10–15 min, achieving over 90% reduction at 60 min. However, adsorption alone contributed minimally to bacterial reduction. Photolysis using UV-C light demonstrated the highest efficiency, reducing *S. aureus* by 60–65% in just 5 min and achieving complete elimination after 90 min. Further research should optimize TiO_2_ concentrations, evaluate film durability, and test real-world applications in hospitals and public areas. Investigating alternative catalyst compositions and light sources could enhance purification efficiency, promoting sustainable air quality improvements.

## 3. Discussions

### 3.1. Kinetic Evaluation

**Figure 7:**
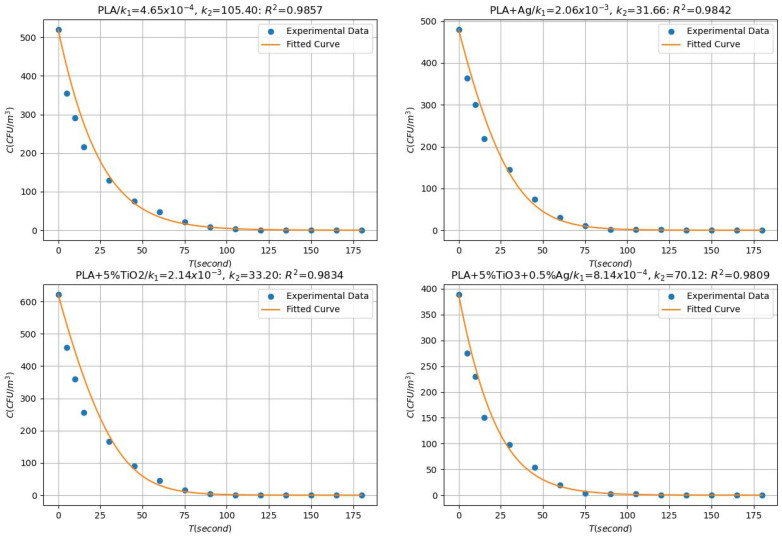
Fitting of the kinetic model through mathematical modeling: photocatalytic oxidation of *S. aureus* with as-developed biofilms.

Incorporating a mathematical model streamlines the photocatalytic oxidation disinfection process, yielding significant time savings. This strategic approach offers a valuable tool for developing improved disinfection strategies to counter the escalating threat of antimicrobial resistance. Figure 7 presents the kinetic evaluation results of the developed biofilms (B1, B2, B3, and B4), revealing a strong correlation between the experimental data and the first-order kinetic equation model. The high R^2^ values (0.9857, 0.9842, 0.9834, and 0.9809 for B1, B2, B3, and B4, respectively) confirm the validity of the first-order kinetic model in describing the photocatalytic processes. A slight variation in R^2^ values indicates the combined hybrid performance of the developed air purifier. Specifically, during photocatalytic oxidation, the process is complemented by activated adsorption and adsorption, which collectively contribute to the disintegration of microbes. The observed trends in bacterial inactivation efficiency can be attributed to the strong correlation between the generation of ROS and the antimicrobial activity, as previously reported by Javier et al. [30]. The calculated first-order (k_1_) and second-order (k_2_) rate constants confirm the role of PCO in the removal of *S. aureus*. The k_1_ values, measured at 4.65 × 10^−4^, 2.06 × 10^−3^, 2.14 × 10^−3^, and 8.14 × 10^−4^ for samples B1, B2, B3, and B4, respectively, highlight variations in bacterial degradation rates based on the specific conditions of the photocatalytic process. A higher k_1_ suggests increased bacterial inactivation efficiency due to enhanced production of ROS and stronger interactions between bacterial cells and the photocatalytic surface. Additionally, the k_2_ values of 105.40, 31.66, 33.20, and 70.12 for B1, B2, B3, and B4, respectively, further emphasize the effectiveness of PCO in bacterial removal. These variations in k_2_ may be linked to differences in TiO_2_ and Ag NPs composition, distribution, or activation within the bioplastic matrix. The observed kinetic parameters demonstrate the efficiency of PCO in *S. aureus* inactivation, reinforcing that the synergistic action of TiO_2_ and Ag NPs enhances bacterial disintegration through ROS-induced oxidative stress. This kinetic analysis provides valuable insights into bacterial degradation dynamics, supporting the potential use of TiO_2_/Ag/PLA biofilms for air purification and antimicrobial surface applications.

### 3.2. Mechanism for Antibacterial Reduction

The antibacterial properties of bioplastic films containing Ag NPs and TiO_2_ result from a combination of chemical and photocatalytic processes that effectively inactivate *S. aureus*. This mechanism involves a series of steps that disrupt bacterial structures and metabolic functions, ultimately leading to cell death. Initially, *S. aureus* adheres to the film surface, facilitated by its unique properties, exposing bacterial cells to embedded antimicrobial agents. Ag NPs then release Ag^+^ ions, which penetrate the bacterial cell wall and membrane, causing structural disruption and increased permeability.

Simultaneously, TiO_2_, upon activation by UV-C light, generates ROS, such as hydroxyl radicals (^•^OH), superoxide (O_2_^−^), and hydrogen peroxide (H_2_O_2_). These ROS exert oxidative stress on bacterial cells, causing lipid peroxidation, protein denaturation, and nucleic acid damage. The synergistic effect of Ag NPs and TiO_2_ amplifies ROS production, accelerating bacterial inactivation and enhancing the overall antibacterial efficiency of the composite material.

**Figure 8:**
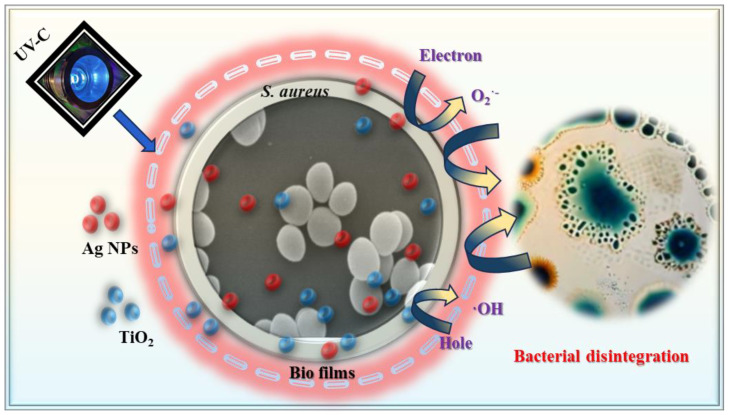
Schematic representation for the PCO mechanism of as-developed B4 biofilm.

The incorporation of TiO_2_ and Ag NPs within the PLA matrix not only enhances the stability of these active agents but also ensures sustained antibacterial activity. The increased generation of ROS serves as the primary mechanism of bacterial disintegration, making the TiO_2_/Ag/PLA bioplastic films highly effective for air purification applications. The proposed pathway of *S. aureus* inactivation is illustrated in Figure 8.

The significance of our findings lies in their potential to inform infection control strategies. Notably, the ability to rapidly achieve high-level disinfection addresses a critical need in healthcare settings, where hospital-acquired infections represent a significant threat.

## 4. Materials and Methods

An experimental study was conducted in the Environmental Health Program at the Faculty of Public Health. The materials and equipment used in this study included polylactic acid (the PLA is derived from renewable resources like corn starch with a grade of 2003D-100%) (derived from renewable sources-NatureWorks), anatase-phase TiO_2_ nanoparticles (Sigma-Aldrich, St. Louis, MO, USA, 10 to 100 nm-100%), silver (Ag) nanoparticles (10–100 nm, Sigma-Aldrich-99.9%), and a Lab Tech model LE20-30/C and LF-250 blown film extrusion machine made in Labtech engineering company LTD. Phraeksa, Thailand.

### 4.1. Preparation of TiO_2_ Infused Bioplastic Films

Bioplastic films were produced using PLA as the primary monomer, chosen for its biodegradable characteristics and potential to replace fossil-based plastics. TiO_2_ with anatase crystals was incorporated into the bioplastic film as a photocatalyst to enhance its photocatalytic properties.

### 4.2. Preparation of TiO_2_ Biofilm Materials

In our previous study, we provided insights into the preparation and characterization of biofilms for benzene removal. The PLA/TiO_2_-anatase composite films (5% by wt. TiO_2_) show high photocatalytic efficiency for benzene degradation, making them promising for indoor air purification [28]. By integrating knowledge from our earlier study with the complexities of bioaerosol systems, this work reinforces previous findings and extends their applicability to a new domain. This study explores photocatalytic oxidation using polylactic acid (PLA) films embedded with Ag nanoparticles and TiO_2_ for bacterial disinfection. Building on this foundation, the current research explores bioaerosol interactions, adapting and refining earlier methodologies. By extending our previous findings, this study offers new perspectives on the hybrid air purifier.

Before fabricating the Ag-TiO_2_/PLA biofilm, the appropriate TiO_2_-to-PLA ratio was evaluated. Various concentrations of TiO_2_, specifically 5%, 10%, and 15% by weight, were blended with PLA and processed into films. The selection of the optimal composition was guided by preliminary assessments, including morphological analysis. Based on these evaluations, the 5% TiO_2_-PLA mixture was identified as the most suitable due to its enhanced structural and morphological characteristics, essential for improving the biofilm’s overall performance.

### 4.3. Preparation of Bio-Composite Materials

PLA pellets were combined with Ag/TiO_2_ in different ratios (Table 1). The mixture was then subjected to melt blending using a twin-screw extruder. The extruder was worked within a temperature range of 100–190 °C and a screw speed of 200 rpm. This process facilitated the uniform distribution of components and the formation of a homogeneous composite material. The resulting composite materials were subsequently processed into bioplastic films using the blown film method.

The composition of the four Formula 1 samples (B1–B4) was carefully tailored to investigate the antimicrobial efficacy of individual materials. Sample B1 served as a control, consisting of 100% PLA without additives. Sample B2 incorporated 0.5% Ag nanoparticles in aqueous solution, leveraging the well-documented antimicrobial properties of Ag NPs. Samples B3 and B4 explored the synergistic effects of combining TiO_2_ with PLA, with the latter also containing 0.5% Ag NPs.

### 4.4. Bioplastic Film as a Photocatalyst

**Figure 9:**
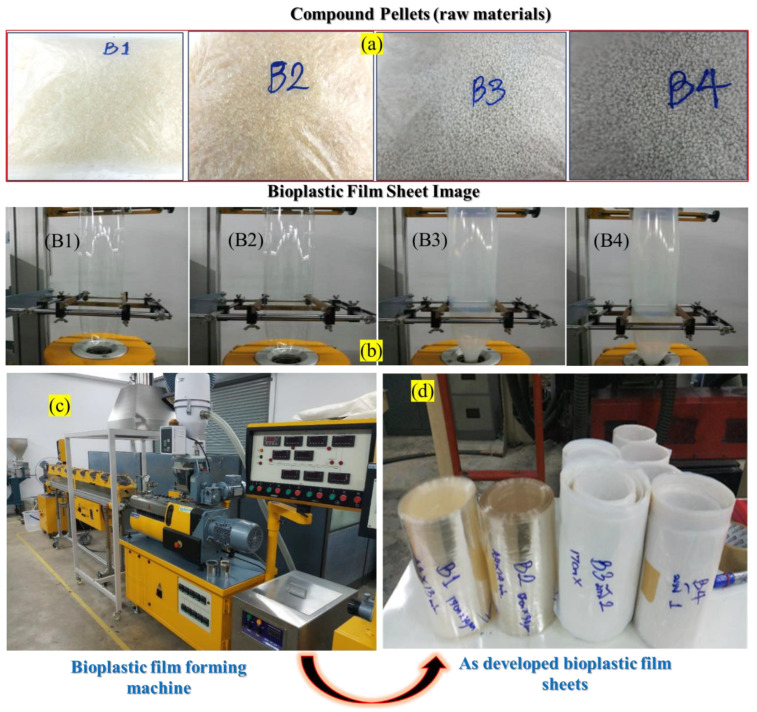
(**a**) Raw materials. (**b**) Blown film extrusion process. (**c**) Real picture of Lab Tech model LE20-30/C and LF-250, and (**d**) as-developed bioplastic films.

The compound pellets formulated according to Table 1 (Figure 9a,b) were subjected to a blown film extrusion process utilizing a specialized machine (Figure 9c) Lab Tech model LE20-30/C and LF-250). The films’ dimensions were carefully controlled, with a specified width of 16 cm and a thickness of 50 microns. The fabricated bioplastic films, shown in Figure 9d, were meticulously collected and stored for subsequent analysis and evaluation.

The blown film extrusion process enabled the production of uniform bioplastic films with consistent geometrical characteristics, a crucial factor in assessing their physical and functional attributes.

### 4.5. Physical Characterization of Bioplastic Films

The surface morphology of the as-developed films was investigated using Scanning Electron Microscopy (SEM) (Model Jx A-840, JEOL-Lab tech, Pleasanton, CA, USA), revealing detailed information about their topographical features.

### 4.6. Design and Construction of Air Purifier

**Figure 10:**
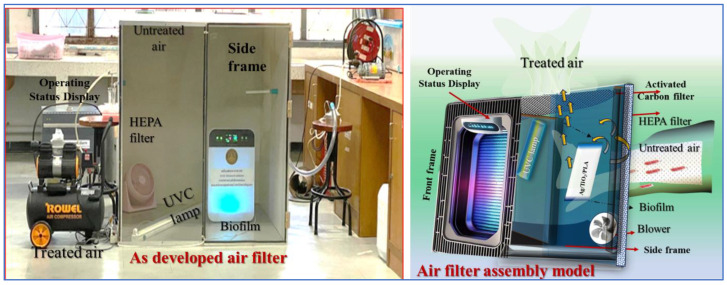
As-developed hybrid air purifier; real vs. schematic representation.

Figure 10 shows the custom-built air purifier, which was designed and constructed for this study, presenting dimensions of 420 mm in width, 210 mm in length, and 476 mm in height (volume ~ 42.02 L). The device had a fan that maintained an airflow rate of 4 L per minute. Internally, the air purifier incorporated the bioplastic film developed in the previous step, a UV-C lamp emitting a wavelength of 254 nanometers, and a slot for inserting the bioplastic film. The UV-C lamp produced a light intensity of 5 mW/cm^2^ at a distance of 1 cm and 10 mW/cm^2^ at a distance of 3 cm. A detailed illustration of the air purifier design is provided in Figure 10.

**Figure 11:**
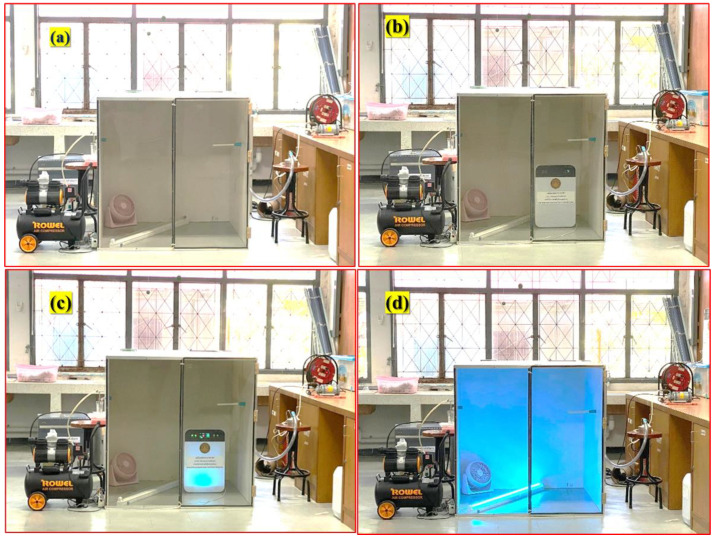
(**a**) Experimental setup, (**b**) experimental setup with air purifier installed, (**c**) experimental setup during the experiment, and (**d**) cleaning the experimental chamber.

A custom-built enclosure was designed with dimensions of 1 m^3^ featuring a hybrid construction of acrylic sheets and plywood. To ensure a sealed environment, rubber gaskets were incorporated into the design. A single fan was integrated into the enclosure to facilitate air circulation. The experimental setup was then housed in a climate-controlled chamber, where temperature and humidity levels were meticulously maintained within a range of 25–27 °C and 40–70% relative humidity, respectively. This specific climatic condition was selected to optimize the efficacy of the air purification process. Subsequently, the air purifier was strategically positioned within the enclosure, with various configurations tested as depicted in Figure 11. The hybrid air purifier incorporates an assembly model that includes a HEPA filter for filtration and an activated carbon layer for adsorption. These components function as a preliminary treatment phase before the PCO process. The HEPA filter efficiently captures airborne particles, while the activated carbon layer helps in trapping gaseous contaminants and volatile organic compounds. This combination enhances the overall purification efficiency. The purifier operates through a three-step process: filtration, adsorption, and photocatalysis to effectively eliminate particulate matter, harmful gases, and microbial pollutants from the air.

### 4.7. Preparation of Bioaerosols

**Figure 12:**
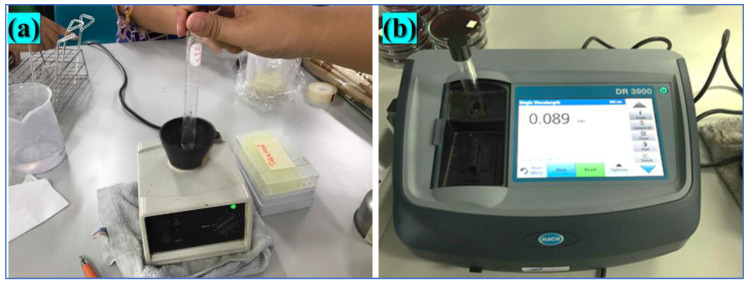
(**a**) Shaking of the substance using a vortex mixer, and (**b**) measurement of absorbance at 600 nm wavelength using a spectrophotometer.

This study was designed to examine the reduction in bioaerosols in the air under various conditions. The experimental procedure consisted of four main sets: control, photolysis, adsorption, and photocatalytic oxidation reaction. Initially, a suspension of *S. aureus* was prepared to simulate bioaerosols using a standardized protocol. The bacteria were first cultured on a PCA agar plate and incubated at 35–37 °C for 18–24 h to ensure optimal growth. A single colony was then selected and resuspended in 10 mL of phosphate-buffered saline (PBS), followed by thorough vortexing to achieve a uniform distribution. The bacterial concentration was standardized by measuring the absorbance at 600 nm using a spectrophotometer, ensuring a target range of 0.08–0.10, which corresponds to approximately 1.5 × 10^8^ CFU/mL. Finally, the suspension was diluted to a concentration of 10^3^–10^4^ CFU/mL, making it suitable for further experimentation. Shaking of the substance using a vortex mixer and measurement of absorbance at 600 nm wavelength using a spectrophotometer are shown in Figure 12.

### 4.8. Experimental Procedure: Investigating Factors Affecting Air Purifier Efficiency

#### 4.8.1. Control Unit

The control unit aimed to study the natural reduction in bioaerosols in the air without an air purifier. The experimental procedure involved spraying a bacterial suspension of 10^3^–10^4^ CFU/mL for 15 min, followed by turning on the fan inside the experimental chamber, then collecting bacterial samples using a single-stage impactor at an airflow rate of 28.3 L per minute, incubating the samples at 35–37 °C for 24–48 h, and, finally, counting colonies and calculating the natural reduction results.

#### 4.8.2. Adsorption Experiment

The adsorption set (adsorption), designed to examine the reduction in bioaerosols using an air purifier equipped with a bioplastic film, involved assembling the air purifier with the bioplastic film but without the UV-C lamp, spraying a bacterial suspension of 10^3^–10^4^ CFU/mL for 15 min, activating the fan and air purifier, collecting bacterial samples via a single-stage impactor, incubating samples at 35–37 °C for 24–48 h, and, finally, counting colonies and calculating the reduction results.

#### 4.8.3. Photolysis Experiment

The photolysis set, designed to investigate the reduction in biological suspended particles using an air purifier equipped solely with UV-C light, consisted of assembling the air purifier without bioplastic film but with the UV-C lamp, spraying a bacterial suspension of 10^3^–10^4^ CFU/m^3^ for 15 min, activating the fan and UV-C lamp, collecting bacterial samples via a single-stage impactor, incubating samples at 35–37 °C for 24–48 h, and, ultimately, counting colonies and calculating the reduction results.

#### 4.8.4. Photocatalytic Oxidation Experiment

The photocatalytic oxidation reaction set, designed to investigate the reduction in bioaerosols using an air purifier equipped with both a bioplastic film and UV-C light, consisted of assembling the air purifier with the bioplastic film and UV-C lamp, spraying a bacterial suspension of 10^3^–10^4^ CFU/mL for 15 min, simultaneously activating the fan, air purifier, and UV-C lamp, collecting bacterial samples via a single-stage impactor, incubating samples at 35–37 °C for 24–48 h, and, ultimately, counting colonies and calculating the reduction results. The kinetic evaluation of the developed photocatalyst has been investigated through the first-order kinetic analysis.

(1) $$\frac{d}{dt}C\left(t\right)=-\frac{k_{1}k_{2}C\left(t\right)}{1+k_{2}C\left(t\right)},$$

where $k_{1}$ the rate constant for bacterial inactivation (dependent on disinfectant concentration and type), the rate constant for bacterial regrowth or recovery is denoted by $k_{2},$ and C(t) is the concentration of bacteria at time t. To determine these constants, we first integrated the kinetic equation with respect to time:

(2) $$\int \frac{1}{C(t)}+k_{2}\;dC=-k_{1}k_{2}\int dt,$$

(3) $$\ln\left|C\right|+k_{2}C=-k_{1}k_{2}t+d,$$

(4) $$t=d-\frac{(\ln\left|C\right|+k_{2}C)}{k_{1}k_{2}},$$

in which *d* is an arbitrary constant. For the initial condition, $C\left(0\right)=C_{0},$ we then will have the fitting equation:

(5) $$t=\frac{1}{k_{1}k_{2}}(\ln\left|\frac{C_{0}}{C(t)}\right|+k_{2}\left(C_{0}-C\left(t\right)\right)\;\;$$

#### 4.8.5. Limitations of This Study

This study used *S. aureus* as a model organism to assess the antimicrobial efficacy of the Ag/TiO_2_/PLA biofilm. While informative, this approach may not fully represent the diversity of indoor air pathogens. Experiments under controlled conditions limit direct real-world applicability, and long-term stability or nanoparticle release was not evaluated. Nevertheless, the biofilm demonstrated strong antimicrobial activity, supporting its potential for future application. Further studies can address environmental variability and safety considerations.

## 5. Conclusions

This study successfully developed an eco-friendly hybrid air purification system utilizing Ag/TiO_2_/PLA biofilms to mitigate indoor air pollution, particularly in high-risk urban environments like Chonburi Province, Thailand, where PM2.5 levels frequently exceed safe limits. This research successfully developed and refined a hybrid air purification system utilizing Ag/TiO_2_/PLA biofilms to tackle the issue of indoor air pollution, particularly in urban areas like Chonburi Province, Thailand, where PM2.5 concentrations often exceed recommended safety limits. By optimizing TiO_2_ concentrations and incorporating Ag nanoparticles, the photocatalytic properties of the PLA-based biofilm were significantly improved. SEM analysis validated the structural and particle distribution in the biofilms. A customized hybrid air purifier model was designed to incorporate Ag/TiO_2_/PLA biofilms into a controlled oxidation system, proving highly efficient in indoor air purification. In particular, the as-developed air purifier functions in adsorption and photocatalytic oxidation. Also, this study emphasized the importance of light intensity (calculated from light-film distance) in optimizing photocatalytic activity, with notable reductions in microbial contamination and airborne pollutants. Compared to traditional adsorption systems, this biofilm-based approach offers a sustainable, energy-efficient, and scalable alternative for improving air quality. The first-order rate constant (k) obtained through modeling helps quantify the efficiency of bacterial disintegration, with higher values indicating faster inactivation. In summary, the Ag/TiO_2_/PLA biofilm system presents a viable and innovative solution for enhancing indoor air quality, particularly in regions with high pollution levels. Its successful integration into air purification systems can significantly mitigate health risks associated with airborne pollutants. Future research ought to focus on evaluating long-term performance, regeneration time for the photocatalyst, and real-world implementation to further advance photocatalytic air purification technologies.

## Figures and Tables

**Table 1:** Composition formulas of as-developed biofilms.

Samples	Composition (Percent Weight)		
	PLA	TiO_2_ (g)	Ag NPs aq. Solution (g)
1(B1)	100	0	0
2(B2)	99.5	0	0.5
3(B3)	95	5	0
4(B4)	94.5	5	0.5

## Data Availability

All data have been included in the manuscript.
